# OPENING PANDORA’S BOX: VIRTUAL UNCOVERING OF GERIATRIC SYNDROMES

**DOI:** 10.1093/geroni/igae098.2928

**Published:** 2024-12-31

**Authors:** Ronan Factora

**Affiliations:** Cleveland Clinic, Cleveland, Ohio, United States

## Abstract

Virtual Capacity Evaluations (VCE) were implemented during the COVID-19 pandemic at the Cleveland Clinic Center for Geriatric Medicine to facilitate Adult Protective Services’ investigations of elder abuse and neglect where a client’s decision making capacity was in question. During the natural process of the evaluation, the unaddressed medical issues were uncovered, specifically identification of cognitive impairment or dementia, gait impairment and falls risk, unexplained weight loss, and non-adherence to medications that increased risk of exacerbation of chronic illness. As many of these persons were also not connected to medical care, these problems were not recognized or managed. In the initial pilot of this program involving 52 individuals, 40 were eventually diagnosed with dementia, 24 had vision impairment, 14 had hearing impairment, 28 were at risk for falls, 24 had unintentional weight loss, and 29 were not taking their medications consistently. Each of these factors potentially limits the person’s ability to function independently, threatens their physical/mental health, and increases their risk of hospitalization and institutionalization. Using the Clinical Frailty Scale (CFS) as an aggregate marker of overall health status, 28 were mildly frail (CFS 5), 14 were moderately frail (CFS 6), and 1 was severely frail (CFS 7). Despite the limited focus and purpose of the VCE, communicating the presence and status of these uncovered medical issues to that person’s medical provider could facilitate evaluation of management of these problems to mitigate their potential impact on that person’s health, functional status, and independence.

